# Synthesis of Fe-CNFs and Mechanistic Insights into Carbon-Water Reaction

**DOI:** 10.3390/nano16110700

**Published:** 2026-06-05

**Authors:** Wenqi Gao, Yuan Meng, Xinran Zhang, Liqiang Liu, Yunjie Zhang, Jin Zhou, Zifei Sun

**Affiliations:** School of Chemistry and Chemical Engineering, Shandong University of Technology, Zibo 255000, China

**Keywords:** Iron-based nanomaterials, carbon gasification, carbon-water reaction, catalytic gasification

## Abstract

Iron-based carbon nanofibers (Fe-CNFs) have garnered significant attention due to their promising applications as functional materials or precursors in the field of catalysis, energy storage, and electromagnetic interference shielding. In this work, electrospun Fe_3_O_4_-CNFs were reduced under a H_2_/Ar atmosphere to obtain Fe-CNFs, and the reduction temperature and holding time were systematically optimized. Notably, a pronounced carbon gasification phenomenon was observed at elevated temperatures (>550 °C), leading to a complete consumption of the carbon matrix. The underlying mechanism was explored using temperature-programmed reduction with mass spectrometry (TPR-MS) and density functional theory (DFT) calculations. The results suggest that the carbon gasification during the H_2_ reduction process is primarily driven by the carbon-water reaction, which can be catalyzed by the in situ-formed Fe nanoparticles. As the temperature increases, various reactions—including hydrogen dissociation, H_2_ spillover, carbon-water reaction, and Boudouard reaction—may progressively consume the carbon framework, ultimately leading to structural collapse and complete material loss. This study elucidates the underlying mechanism of carbon-water reaction and provides practical guidance for the optimization of synthesis parameters, thereby enhancing the yield and structural integrity of free-standing Fe-CNFs for their application in catalysis and energy storage-related fields.

## 1. Introduction

Iron-based nanomaterials (e.g., Fe, Fe_3_O_4_, Fe_2_O_3_) have attracted extensive attention due to their excellent magnetic properties, catalytic activity, electrochemical performance, and low cost [1,2,3,4]. They have been widely explored as active materials or precursors to apply in the field of energy conversion, environmental remediation, electromagnetic shielding, biomedicine, and advanced catalytic systems, etc. [1,5,6,7,8,9]. Among them, Fe^0^-based carbon nanofibers (Fe-CNFs), in which zero-valent iron nanoparticles are embedded within a carbon matrix, have demonstrated broad applicability across multiple domains, including energy storage, algae removal, pollutant degradation, catalysis, etc. [10,11,12,13].

Electrospinning has become one of the most powerful and versatile methods for fabricating free-standing nanofibrous membranes owing to its operational simplicity, tunable morphology, and structural uniformity [14,15,16]. Such architectures effectively overcome the common drawbacks of powdered materials such as agglomeration, detachment, and poor electrical contact, thereby offering new opportunities for the development of free-standing and scalable Fe-based functional materials. Free-standing Fe-CNFs can be synthesized via H_2_ reduction in electrospun Fe_3_O_4_-CNFs. During the reduction process, the thermal treatment temperature and holding time play critical roles in determining the phase evolution of the electrospun Fe_3_O_4_-CNFs. The reduction behavior of Fe_3_O_4_ nanoparticles by H_2_ has been investigated in previous studies. As demonstrated by Liu et al., the reduction in Fe_3_O_4_ by H_2_ can proceed via two coexisting pathways—a direct one-step route (Fe_3_O_4_ → Fe) and a sequential two-step route (Fe_3_O_4_ → FeO → Fe)—with the dominant pathway depending on the reaction temperature, as revealed by in situ environmental transmission electron microscopy [17]. Given that CNFs exhibit a higher specific surface area than nanoparticles, the enhanced interfacial contact with hydrogen is likely to affect the reduction behavior, thereby leading to variations in reduction time and reduction temperature relative to Fe_3_O_4_ nanoparticles. However, the influence of H_2_ on the phase evolution of electrospun Fe_3_O_4_-carbon nanofibers (Fe_3_O_4_-CNFs), to the best of our knowledge, remains unexplored. Given these differences and the wide application of Fe-CNFs, a systematic investigation into the effects of temperature and holding time on the reduction behavior of Fe_3_O_4_-CNFs is essential.

Beyond the phase evolution of Fe_3_O_4_ during reduction, another critical concern arises from the thermal stability of the carbon matrix itself. Carbon materials are known to undergo complete gasification at elevated temperatures under H_2_ atmosphere, a process that may be accelerated in the presence of a catalyst [18]. Existing studies have predominantly focused on carbon powder or coke, with limited attention devoted to the behavior of electrospun CNFs under H_2_ atmosphere. Given the enhanced surface area and three-dimensional network structure of electrospun CNFs, the gasification behavior of such materials may deviate considerably from that of carbon powder or coke. However, the mechanism underlying catalyst-assisted CNFs gasification remains scarcely investigated. Specifically, the processing conditions, such as temperature and holding time, under which the carbon matrix can be preserved, remain poorly understood.

Carbon gasification, in this context, refers to the progressive consumption of the carbon framework by various gaseous species, including H_2_O, H_2_, and CO_2_ at elevated temperatures, ultimately leading to severe mass loss or even complete disappearance of the carbon matrix. This phenomenon is of particular concern during the synthesis of metal–carbon composite nanofibers, as the incorporation of metal species may influence the extent of gasification and compromise the structural integrity of the final product. Therefore, elucidating the temperature-dependent gasification behavior and its underlying mechanism would be essential for identifying feasible processing parameters.

In our initial attempts to prepare Fe-CNFs via H_2_ reduction, we observed that the carbon matrix largely/completely disappeared when the temperature was higher than 550 °C. This observation motivated us to systematically investigate the gasification behavior on the iron-based CNFs, a recurring yet insufficiently understood phenomenon in the synthesis of metal–carbon composites.

In this study, free-standing Fe-CNFs were synthesized via the reduction of electrospun Fe_3_O_4_-CNFs in a H_2_/Ar atmosphere. The effects of reduction temperature and holding time were systematically investigated, and the optimal conditions were identified. The mechanism of carbon gasification was explored by temperature-programmed reduction mass spectrometry (TPR-MS) and density functional theory (DFT) calculations. The results suggest that the water-carbon reaction may be the main reaction for carbon gasification. Based on the analysis of thermodynamic data and DFT calculations, the formation of CO and H_2_ from carbon-water reaction is thermodynamically favorable. High temperature can help the formation of the transition state during carbon gasification, and Fe likely plays a necessary catalytic role during gas desorption.

## 2. Materials and Methods

### 2.1. Chemicals and Reagents

Iron(III) acetylacetonate (Fe(acac)_3_,98%), Polyacrylonitrile (PAN, average Mw 149,000–151,000), and N,N-dimethylformamide (DMF, 99.9%) were purchased from Shanghai Aladdin Biochemical Technology Co., Ltd., Shanghai, China. In total, 5% hydrogen (H_2_)/argon (Ar) and high-purity Ar gas were purchased from Shandong Baiyan Chemical Co., Ltd., Zibo, China.

### 2.2. Synthesis of Fe-CNFs Materials

#### 2.2.1. Electrospinning

First, 0.3750 g PAN was dissolved in 4.3125 g DMF at 60 °C. Subsequently, 0.5625 g of iron(III) acetylacetonate (Fe(acac)_3_) was added to the PAN solution and stirred overnight to obtain a homogeneous solution. Then, 10 mL of the obtained solution was loaded into a syringe equipped with a stainless-steel needle. Electrospinning was carried out at 20 kV, and a solution feed rate was 0.8 mL h^−1^ using aluminum (Al) foil as the collector. The distance between the tip of the needle and the Al foil was set to 20 cm. After electrospinning, the precursor membrane was peeled off the Al foil.

#### 2.2.2. Stabilization and Carbonization

The as-spun precursor membrane was stabilized at 220 °C for 1 h in air before cooling it to room temperature. Then the as-prepared sample was transferred to a tube furnace (quartz tube: o.d. 50 mm, i.d. 43 mm, heating zone 200 mm) and carbonized at 600 °C at a ramp rate of 2 °C min^−1^ under Ar atmosphere with a flow rate of 50 sccm to obtain Fe_3_O_4_-based nanofibers.

#### 2.2.3. H_2_ Reduction

The Fe_3_O_4_-CNFs membrane was placed in a ceramic boat in a tube furnace. The sample was heated to the target temperature (550 °C/600 °C/650 °C) under 5% H_2_/Ar mixed gas at a flow rate of 45 sccm and held for 1–2 h. The samples prepared at different reduction temperatures are summarized in Table S1.

### 2.3. Materials Characterization

#### 2.3.1. Scanning Electron Microscopy (SEM)

The microstructure and morphology of the nanofibers were characterized using Field Emission Scanning Electron Microscopy (FE-SEM, SIRION 200, Eindhoven, The Netherlands).

#### 2.3.2. X-Ray Diffraction (XRD)

The crystal structure was characterized by X-ray diffraction using Cu Kα radiation (λ = 0.15418 nm) operated at 40 kV and 35 mA over a 2θ range of 10–80° (XRD, Bruker AXS D8 Advance, Karlsruhe, Germany). The XRD measurements were primarily employed to identify the chemical composition of the as-prepared iron-based carbon nanocomposites.

#### 2.3.3. Fourier-Transform Infrared Spectroscopy (ATR-FTIR)

The functional groups and the successful synthesis of the nanofibers were analyzed by attenuated total reflectance Fourier-transform infrared spectroscopy (ATR-FTIR, Nicolet iS50, Thermo Fisher Scientific, Waltham, MA, USA) over the range of 2000–450 cm^−1^.

#### 2.3.4. Temperature-Programmed Reduction Mass Spectrometry (TPR-MS)

The process of reduction by H_2_ was explored by temperature-programmed reduction (TPR). Approximately 50 mg of the ground sample was used in each run. The samples were first dehydrated under Ar at 100 °C for 1 h and then cooled down to room temperature. A mixture of 5% H_2_ in Ar (45 mL/min) was then switched into the system, and the sample was heated up to 800 °C from room temperature at a rate of 10 °C/min and held for 1 h before cooling down to room temperature. The amount of H_2_ change and the produced gases during the reduction were monitored by a mass spectrometer (MS, Cirrus 2, MKS Spectra Product, Milpitas, CA, USA).

#### 2.3.5. Transmission Electron Microscopy (TEM)

The morphology and microstructure of Fe_3_O_4_-CNFs were examined by transmission electron microscopy (Talos F200i, Thermo Fisher Scientific, Waltham, MA, USA).

#### 2.3.6. Brunauer–Emmett–Teller (BET) Analysis

Specific surface area and pore size of the Fe_3_O_4_-CNFs were detected by N_2_ adsorption–desorption isotherms at 77 K on a Micromeritics 3Flex.

### 2.4. Computational Details

All quantum chemical calculations were performed using the Gaussian 16 program package based on DFT [19]. Geometry optimizations of the reactants, transition states, and products were carried out at the B3LYP/6-31G* level of theory. The optimized structures and transition states were further subjected to single-point energy corrections using the M062X functional with the def2-TZVP basis set with D3 dispersion correction. This article mainly conducts the noncatalytic reaction path.

## 3. Results and Discussion

**Figure 1:**
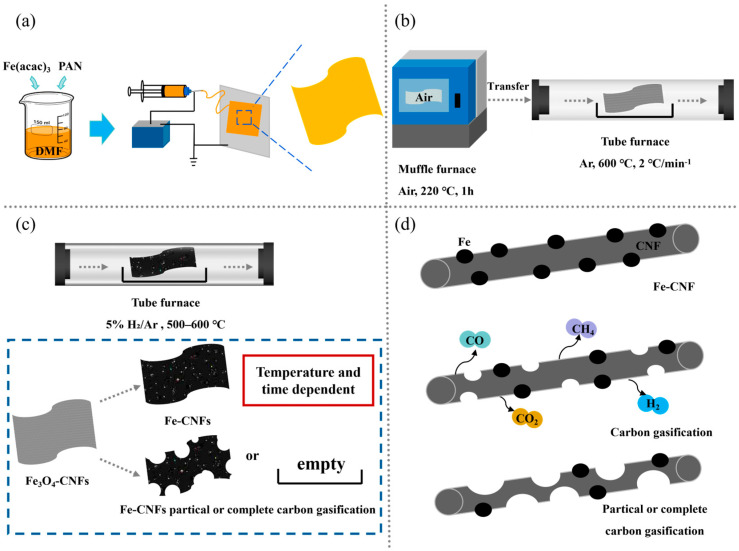
Schematic illustration of the synthesis of Fe-CNFs and the carbon gasification process. (**a**) Electrospinning. (**b**) Stabilization in air and carbonization in Ar in a tube furnace (quartz tube: o.d. 50 mm, i.d. 43 mm, heating zone 200 mm) to obtain Fe_3_O_4_-CNFs. (**c**) H_2_ reduction in the same tube furnace (**d**) Scheme of the Fe-catalyzed carbon gasification process.

**Figure 2:**
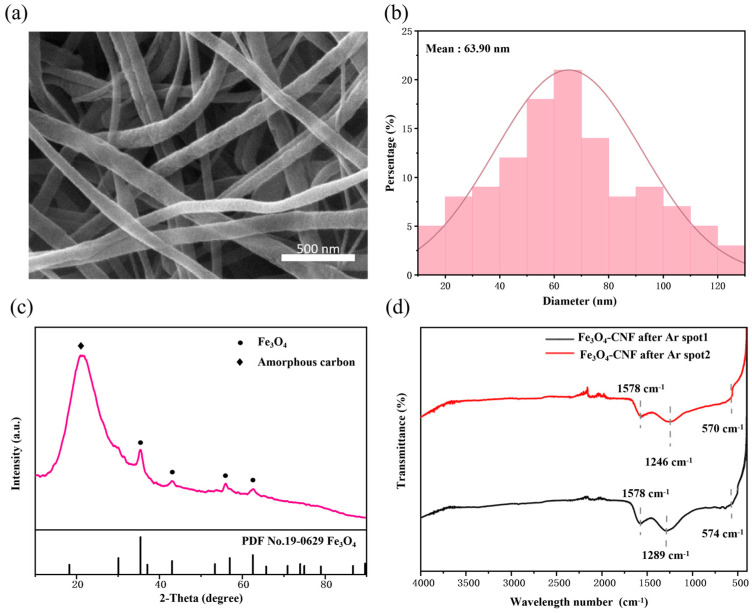
(**a**) SEM images of Fe_3_O_4_-CNFs; (**b**) size distribution of Fe_3_O_4_-CNFs; (**c**) XRD pattern; and (**d**) IR spectra of Fe_3_O_4_-CNFs.

**Figure 3:**
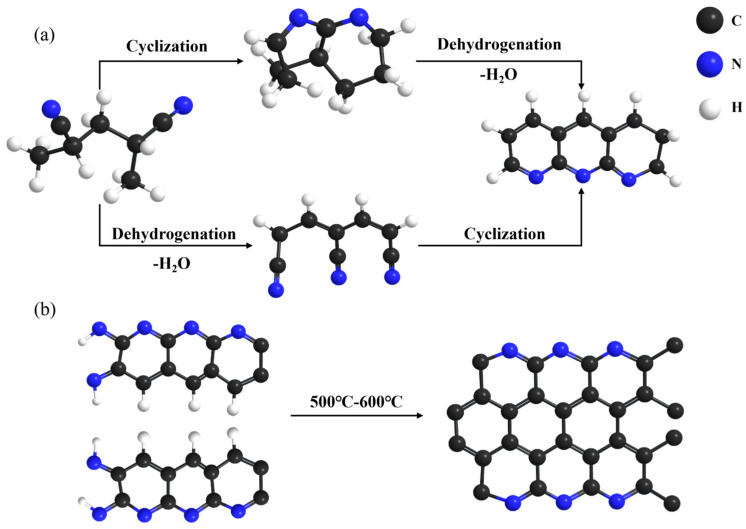
Schematic illustration of the chemical transformations during (**a**) PAN stabilization, (**b**) the carbonization of PAN precursor.

The step-by-step synthetic route of Fe-CNFs is illustrated in Figure 1. In short, PAN/Fe(acac)_3_ precursor was electrospun to form a nanofiber membrane (Figure 1a). The as-prepared membrane was then stabilized in air and carbonized in Ar to yield Fe_3_O_4_-CNFs (Figure 1b) before reducing to metallic Fe^0^-CNFs under H_2_/Ar atmosphere (Figure 1c). However, when the reduction temperature of H_2_/Ar is higher than 550 °C, carbon materials start to gasify. The scheme of reactions occurring during H_2_ reduction is demonstrated in Figure 1d. The morphology of Fe_3_O_4_-CNFs was explored by SEM. As shown in Figure 2a,b, the sample exhibits a well-defined three-dimensional interconnected nanofibrous network. The average diameter of the fiber was 63.9 nm. The TEM image shows the size range of the Fe_3_O_4_ nanoparticle is 21 to 31 nm with an average diameter of 27.85 nm (Figure S5). Figure 2c shows the crystallographic structure of the electrospun samples after carbonization. The diffraction peak at approximately 22° corresponds to amorphous carbon, while all the other peaks are attributed to Fe_3_O_4_. The absence of impurity indicates the phase purity of the as-synthesized Fe_3_O_4_-CNFs. Figure 2d displays the Fourier-transform infrared (FT-IR) spectra collected from two different regions of the same Fe_3_O_4_-CNF membrane. These two spectra show nearly identical peak positions and intensities, demonstrating the uniform chemical composition and homogeneous dispersion of Fe_3_O_4_ throughout the entire nanofiber film. The characteristic absorption band at around 570 cm^−1^ is assigned to the Fe–O stretching vibration of Fe_3_O_4_. The prominent band at 1578 cm^−1^ corresponds to the overlapping stretching vibrations of C=C and C=N bonds, which is indicative of the formation of a conjugated sp^2^ carbon network derived from the cyclization and aromatization of the PAN precursor. The weaker band observed near 1246 cm^−1^ can be attributed to C–N stretching vibrations within the cyclized carbon backbone, suggesting the retention of nitrogen-containing heterocyclic structures during carbonization [20,21,22,23,24]. These FT-IR results further confirm the successful synthesis of Fe_3_O_4_–CNFs without forming any impurity. Based on the previous study elsewhere, the changes in the microstructure of PAN during pyrolysis are as follows: PAN underwent dehydrogenation and cyclization reactions during stabilization in air, resulting in the formation of a ladder-type structure (Figure 3a). The generation of double bonds and unsaturated bonds enhanced the thermal stability of the polymer and mitigated chain scission during subsequent carbonization. During carbonization, the stabilized structure underwent further aromatic growth and polymerization (Figure 3b) [25].

**Figure 4:**
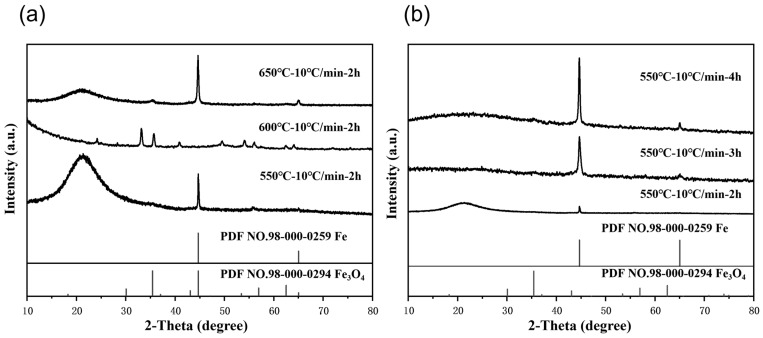
XRD pattern of reduced samples with different (**a**) temperatures and (**b**) holding times.

To illustrate the optimal reduction conditions for the synthesis of Fe-CNFs, the effects of temperature and holding time on the phase evolution of iron-based CNFs were investigated by XRD. As shown in Figure 4a, under the same ramping rate and holding time (2 h), the sample reduced at 550 °C exhibited a distinct Fe peak at 44.7° corresponding to the (110) plane of α-Fe, whereas the Fe_3_O_4_ peak was barely detectable. In contrast, the samples reduced at 600 and 650 °C showed observable Fe_3_O_4_ peaks or other impurity peaks, indicating that higher reduction temperatures promoted the formation of undesirable material. Therefore, 550 °C was selected as the optimal temperature to investigate the effect of holding time. As shown in Figure 4b, a gradual increase in the intensity of the Fe peak was observed with prolonged holding time. Holding times of 3 and 4 h both yielded distinct Fe peaks, with no detectable impurities. For the sake of saving time and energy, a holding time of 3 h was considered compared with 4 h. Notably, it was observed that when the reduction temperature reached 550 °C or higher, the samples were subject to partial or complete carbon gasification (Figure S1), with higher temperatures increasing the possibility of carbon gasification.

**Figure 5:**
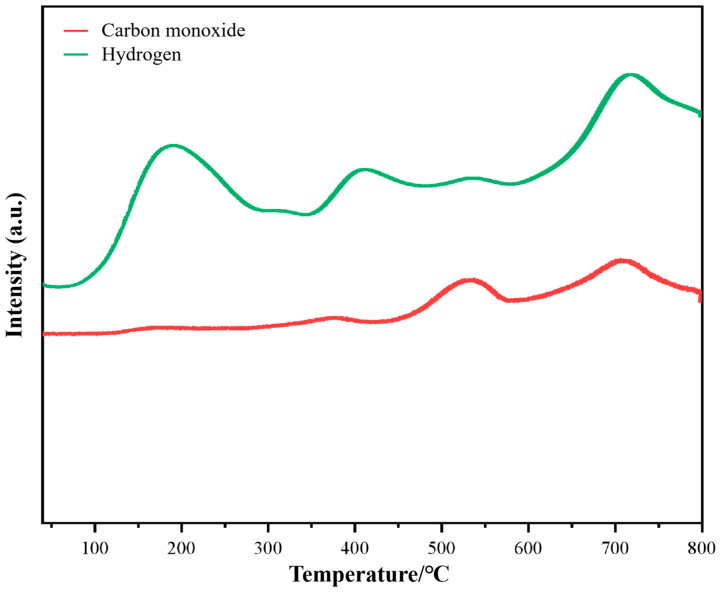
TPR-MS analysis of Fe_3_O_4_-CNFs.

To investigate the mechanism of carbon gasification during H_2_ reduction, TPR-MS was employed to monitor hydrogen consumption and gas evolution as a function of temperature. As shown in Figure 5, TPR-MS data show four distinct hydrogen peaks at approximately 188, 408, 530, and 710 °C. To elucidate the potential reactions associated with these four peaks, we propose the following hypothesis. The peak at 188 °C may be attributed to the hydrogen desorption from the carbon materials. Although BET analysis (Figure S3) indicates powdered Fe_3_O_4_-CNFs is not a porous material, its membrane, composed of a 3D interconnected nanofibrous network, contains numerous inter-fiber voids and interstitial spaces, which can facilitate the hydrogen physisorption [26,27]. Moreover, the in situ formed Fe nanoparticles might enhance H_2_ adsorption due to the hydrogen spillover effect [28]. During the H_2_ reduction process, H_2_ molecules may first dissociate on the surface of the Fe nanoparticle, generating active atomic hydrogen that subsequently migrates and stabilizes at the adjacent carbon defects, edges, or micropores. As the temperature increases to approximately 188 °C, chemically adsorbed hydrogen begins to desorb due to thermally induced bond cleavage, and atomic hydrogen recombines to form H_2_, producing a distinct desorption signal of H_2_ in the TPR-MS profile. With further temperature increases, Fe_3_O_4_–CNFs are reduced to Fe–CNFs and produce water as one of the products at a faster speed. As shown in Table 1, Reaction No. 1 and No. 2 are both H_2_ reduction in Fe_3_O_4_. Reaction No. 1 is thermodynamically allowed when T > −192 °C, which means it can occur across all the temperature ranges we studied, whereas Reaction No. 2 can only happen at T > 626.7 °C. At 408 °C, Reaction No. 1 is thermodynamically favorable. Water in the condensed phase may be produced, but it vaporizes to the gaseous phase immediately. The generated gaseous water can participate in the carbon gasification reaction No. 5, and the produced CO_2_ may further participate in reaction No. 6 to produce CO. This produced CO, which could then be adsorbed on Fe, thereby accounting for the absence of a CO peak at 408 °C [29,30,31].

When the temperature reaches approximately 530 °C, the carbon-water reaction (Reaction No. 4) may become dominant, leading to the production and release of CO and H_2_. Concurrently, the CO previously adsorbed at approximately 408 °C is also released as the temperature increases. The observed decrease in the reaction temperature relative to the theoretical value may be attributed to the catalysis effect of Fe, which might effectively lower the energy barrier of the carbon-water reaction by weakening C–C bonds and facilitating oxygen transfer. In addition, the strong CO signal detected at 530 °C may also be from the Boudouard reaction (Reaction No. 3). The catalysis effect of Fe may lower the actual onset temperature of the Boudouard reaction in this case [32]. As shown in Figure S2, the CO_2_ signal begins to decrease at approximately 350 °C, accompanied by a corresponding rise in the CO signal. This trend suggests that CO_2_ generated from Reaction No. 5 may be consumed via the Fe-catalyzed Boudouard reaction (Reaction No. 3), and the released CO will contribute to the CO signal detected at 530 °C. After further heating to ~710 °C, the temperature exceeds the thermodynamic threshold for Reaction No. 2. At ~710 °C, the reduction in Fe_3_O_4_ by H_2_ proceeds via a pathway that directly generates gaseous H_2_O. Without the need to vaporize, gaseous H_2_O can participate in Reaction No. 4 more rapidly. As a result, the mass spectrum exhibits pronounced CO and H_2_ peaks at 710 °C. Although CH_4_ and CO_2_ are also generated during H_2_ reduction, their signals are at negligible levels compared to those of H_2_ and CO. The low CO_2_ signal aligns with its consumption through the aforementioned Boudouard reaction, indicating that the reaction pathways yielding CH_4_ and CO_2_ as final products are of minor significance under the present experimental conditions.

**Figure 6:**
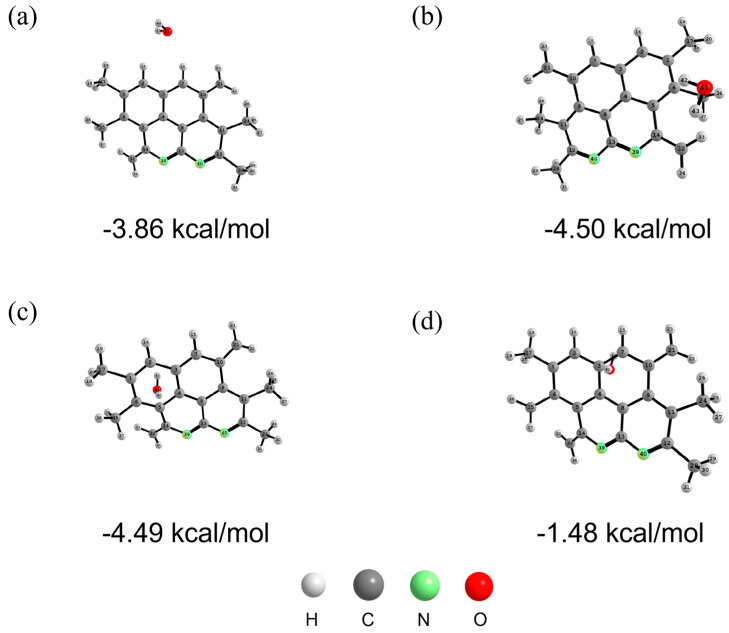
Optimized adsorption geometries and corresponding adsorption energies of hydroxide at diverse active sites on N-doped carbon. Panels (**a**–**d**) correspond to C7, C6, C5 and C3 sites, respectively: (**a**) C7 (−3.86 kcal/mol); (**b**) C6 (−4.50 kcal/mol); (**c**) C5 (−4.49 kcal/mol); (**d**) C3 (−1.48 kcal/mol).

To gain more insights into the mechanism of the carbon-water reaction, a possible reactive pathway was investigated using DFT calculations. Compared with CO, the concentration change in CO_2_ is negligible (Figure S2). Therefore, the reaction that is not related to CO_2_, i.e., Reaction No. 4, was selected for computational investigation. First, the adsorption of H_2_O at different sites on the carbon surface was examined. As illustrated in Figure 6, the most stable adsorption configuration for H_2_O is the surface site depicted in Figure 6b. The reactive pathway was then based on this configuration. When H_2_O is absorbed on the surface carbon, a transition state containing the structure of C···O (TS1) may gradually form, and the effective energy barrier of this step is 1.41 eV. This step is endothermic, consistent with the experimental requirement of heating at high temperature. In the second transition state (TS2), the energy barrier for hydrogen desorption is 2.47 eV, which is relatively high for a non-catalytic pathway. (Figure S4) The DFT results indicate that Reaction No. 4 is kinetically hindered and unlikely to occur without the presence of a catalyst. However, the TPR-MS results clearly detected the evolution of H_2_ and CO, confirming that the gas desorption did proceed. Given that Fe is a widely used and versatile metal catalyst [13,32,33,34], the discrepancy between the DFT prediction and the experimental observation suggests that the in situ formed Fe nanoparticles play a catalytic role in lowering the energy barrier of the hydrogen desorption. Taken together, the DFT and TPR-MS results indicate that Reaction No. 4 is possible to occur with the presence of Fe as the catalyst.

## 4. Conclusions

In this study, electrospun Fe_3_O_4_–CNFs were fabricated and reduced under 5% H_2_/Ar to obtain free-standing Fe–CNFs. The evolution of crystallinity was found to strongly depend on the combined effect of reduction in temperature and holding time. When the reducing temperature is higher than 550 °C, the phenomenon of carbon gasification starts to occur. The mechanism of carbon gasification during H_2_ reduction in Fe_3_O_4_ was investigated experimentally and theoretically. Based on the TPR-MS analysis, the carbon gasification phenomenon might primarily be from the reaction between water and carbon with the presence of Fe as a catalyst. Reaction No. 4 is likely the main reaction among all water-carbon reactions. DFT calculates the possible reactive pathway of reaction No. 4 and confirms that the catalyst, i.e., Fe, is necessary for the gas desorption. To the best of our knowledge, this is the first time to explore the mechanism for water-carbon reaction on electrospun Fe-CNFs. Our findings provide guidance for optimizing the experimental conditions when synthesizing catalyst-containing CNFs.

## Figures and Tables

**Table 1:** Thermodynamic parameters for the hypothesized reactions involved in the carbon gasification process during the H_2_ reduction in Fe_3_O_4_-CNFs.

No.	Reaction	Temperature Condition (ΔG < 0)
1	Fe_3_O_4_ (s) + H_2_ (g) → Fe (s) + H_2_O (l)	T > −192 °C
2	Fe_3_O_4_ (s) + H_2_ (g) → Fe (s) + H_2_O (g)	T > 626.68 °C
3	C (s) + CO_2_ (g) → CO (g)	T > 704.18 °C
4	C (s) + H_2_O (g) → CO (g) + H_2_ (g)	T > 582 °C
5	C (s) + H_2_O (g) → CO_2_ (g) + H_2_ (g)	T > 529 °C
6	CO_2_ (g) + H_2_ (g) → CO (g) + H_2_O (l)	T > −235.72 °C

## Data Availability

The original contributions presented in this study are included in the article/Supplementary Materials. Further inquiries can be directed to the corresponding authors.

## Supplementary Materials

The following supporting information can be downloaded at: https://www.mdpi.com/article/10.3390/nano16110700/s1, Figure S1: Photograph of samples subjected to carbon gasification; Figure S2: TPR-MS profiles for CH_4_, CO_2_, and CO detected during H_2_ reduction; Figure S3: N_2_ adsorption–desorption isotherms and the corresponding pore size distribution of Fe_3_O_4_-CNFs; Figure S4: Proposed reaction pathway for the interaction of H_2_O with the carbon surface; Figure S5: TEM image of electrospun Fe_3_O_4_-CNFs; Table S1: Summary of the Fe-CNFs samples prepared under different reduction temperatures and holding times.
