# Supplementary material for: Synthesis of Fe-CNFs and Mechanistic Insights into Carbon-Water Reaction
Source: Nanomaterials (Basel). 2026 Jun 5;16(11):700. doi: 10.3390/nano16110700 (PMC13258609; doi:10.3390/nano16110700)
Supplement: Supplementary file 1 [file nanomaterials-16-00700-s001.zip › nanomaterials-4269114-supplementary.pdf]

Supporting Information

Synthesis of Fe-CNFs and Mechanistic Insights into Carbon-Water Reaction

Wenqi Gao†, Yuan Meng†, Xinran Zhang, Liqiang Liu, Yunjie Zhang, Jin Zhou and Zifei Sun**

[1]College of Chemistry and Chemical Engineering, Shandong University of Technology, Zibo, Shandong 255000, People's Republic of China

[#]W. Gao and Y. Meng contributed equally to this work

[*]Corresponding author

zhoujin@sdut.edu.cn, sunzifei@sdut.edu.cn

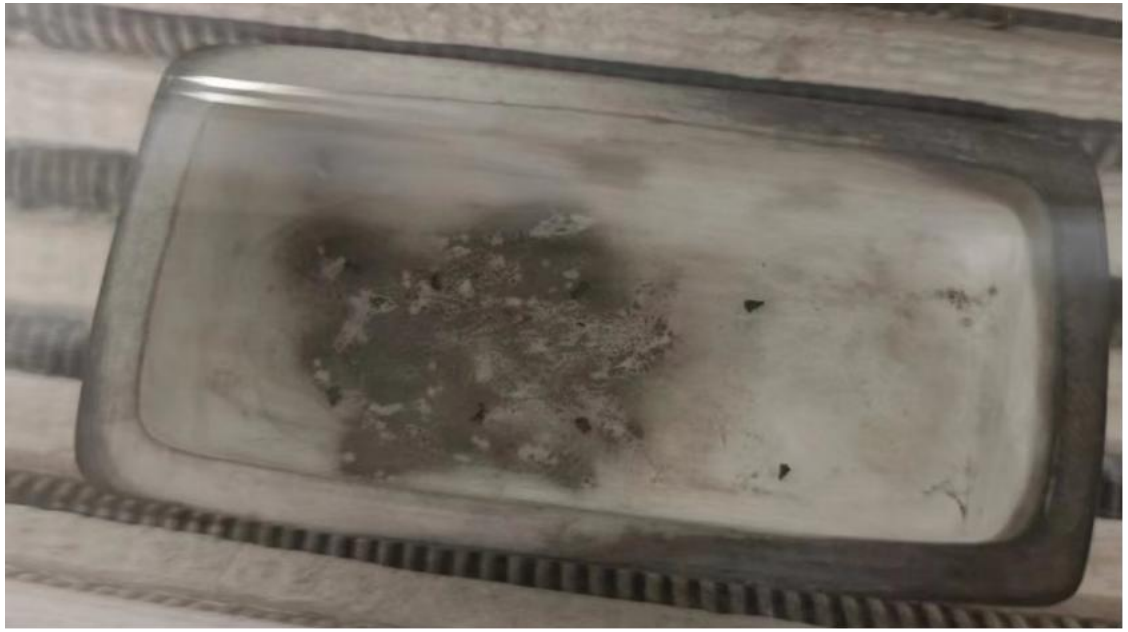

Figure S1: Photograph of samples subjected to carbon gasification

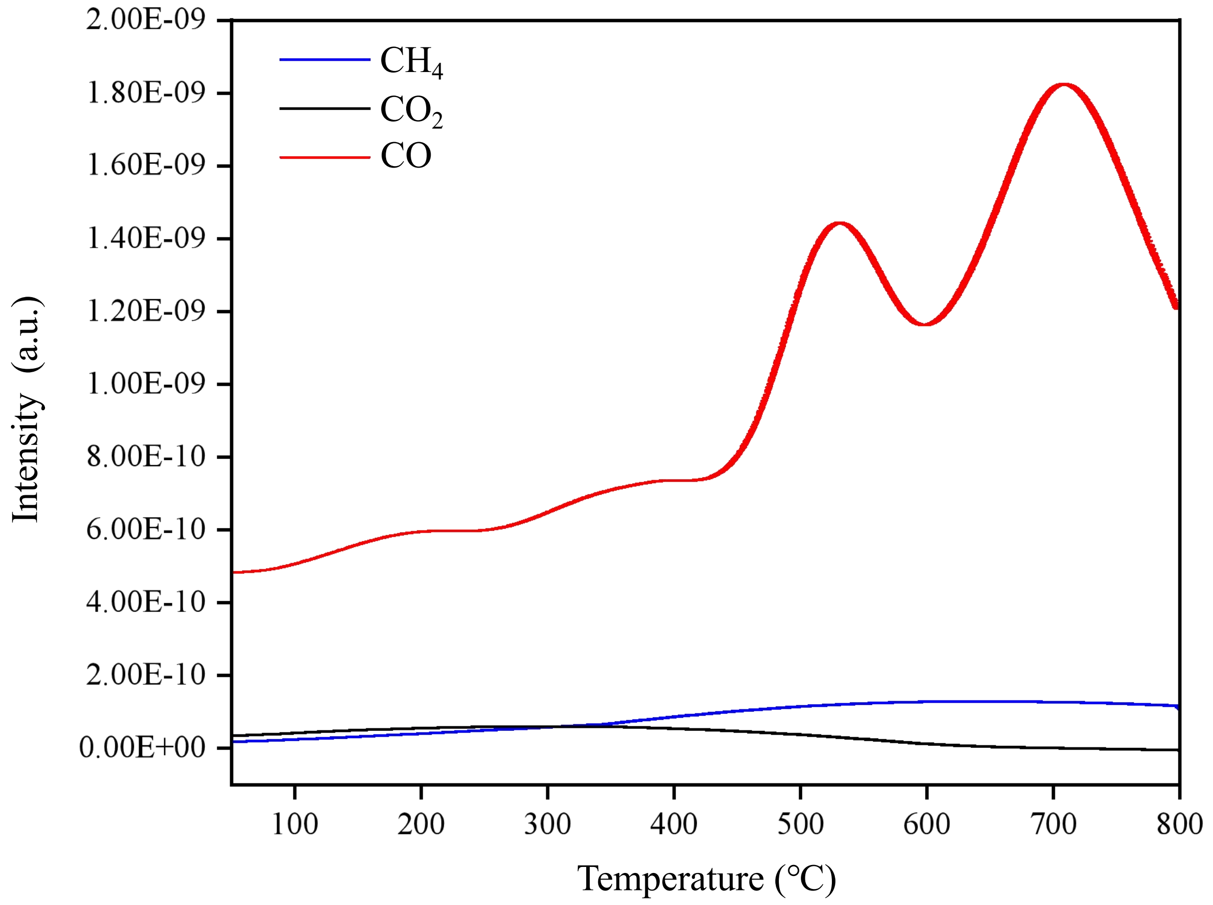

Figure S2: TPR-MS profiles for CH_4, CO_2 and CO detected during H_2 reduction.

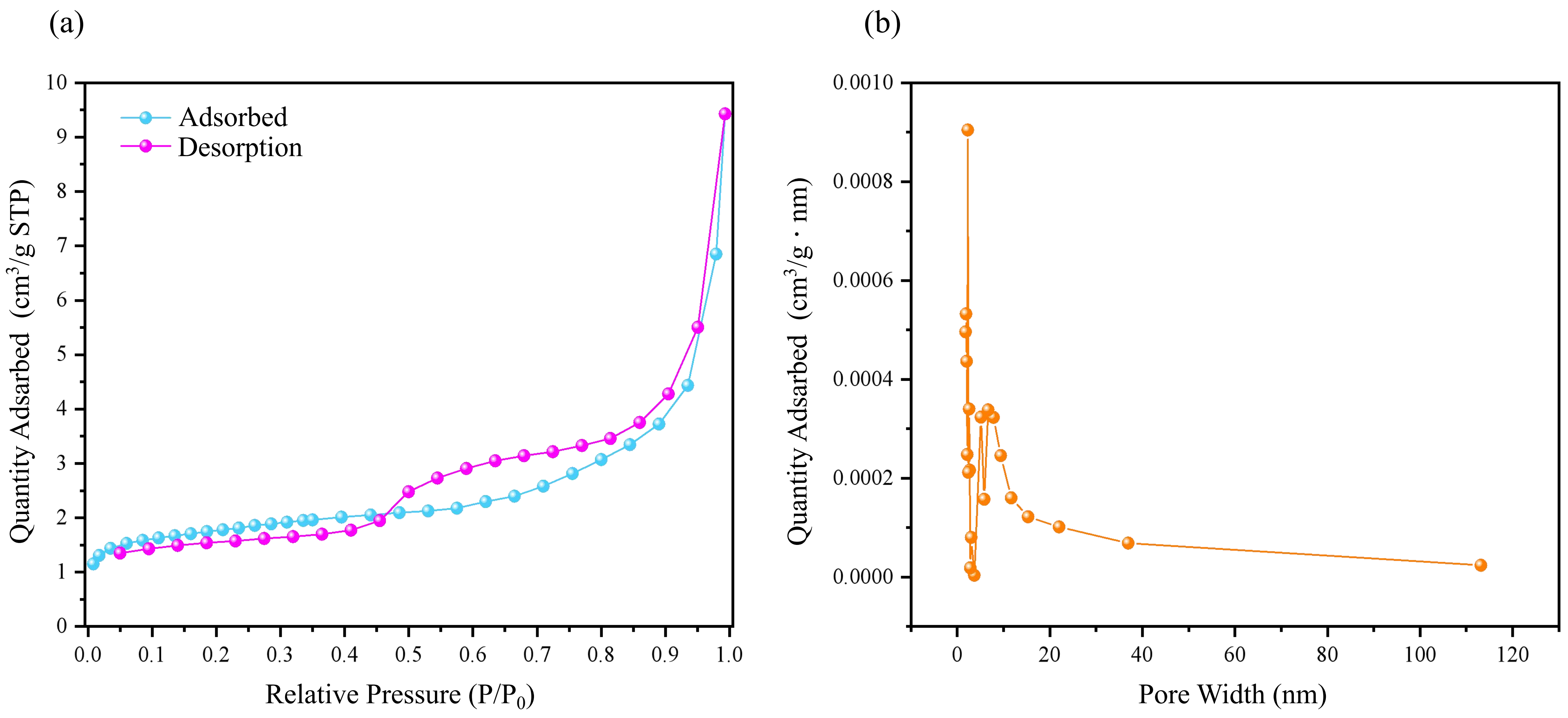

Figure S3: N_2 adsorption-desorption isotherms and the corresponding pore size distribution of Fe_3O_4-CNFs

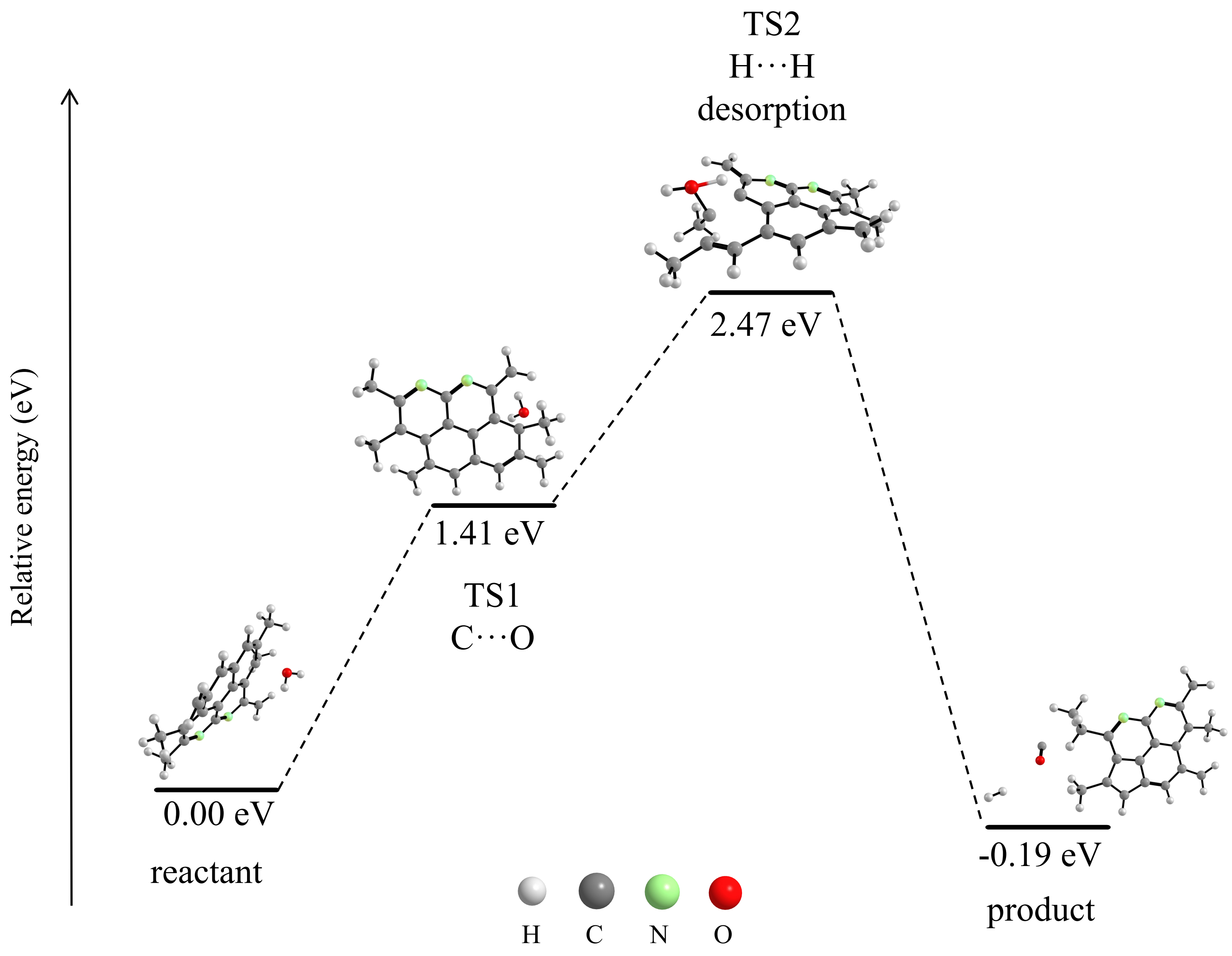

Figure S4: Proposed reaction pathway for the interaction of H_2O with the carbon surface.

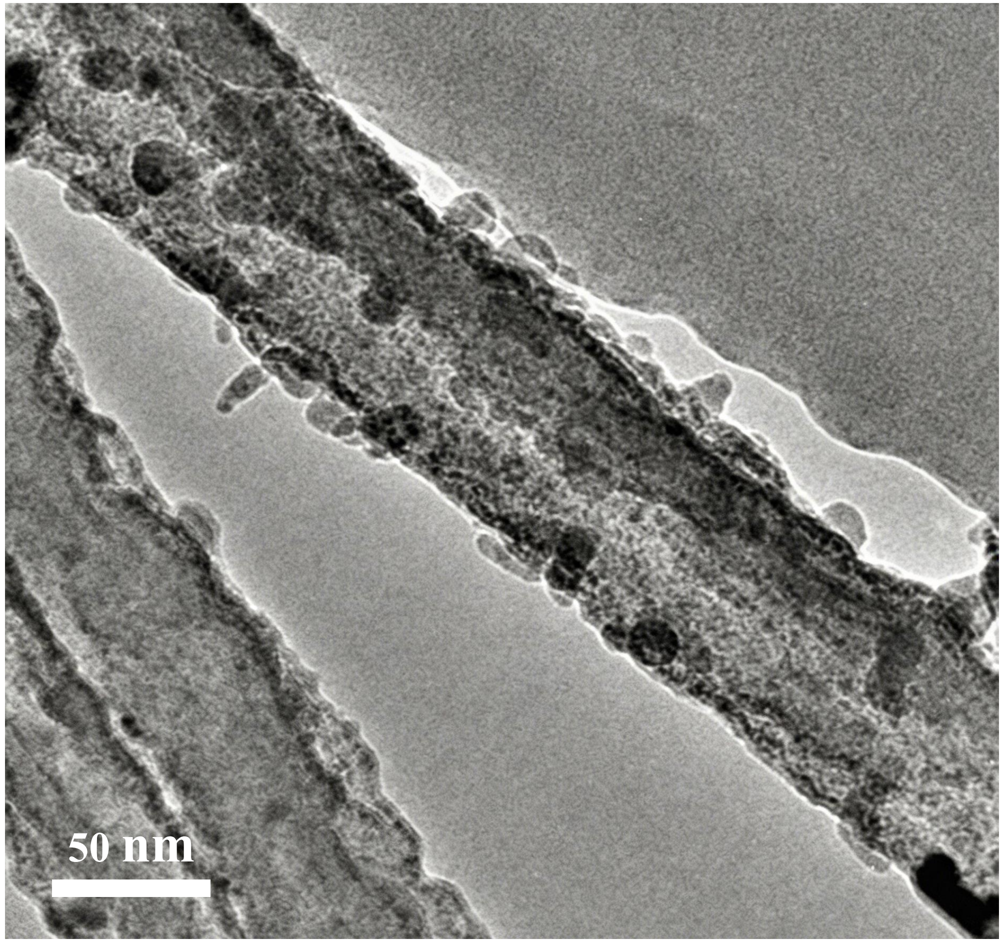

Figure S5:TEM image of electrospun Fe_3O_4-CNFs.

Table S1: Summary of the Fe-CNFs samples prepared under different reduction temperatures and holding times.

**Sample ID**	**Temperature (°C)**	**Holding time (h)**	**Atmosphere**
Fe-CNFs-550-2	550	2	5% H_2/Ar
Fe-CNFs-550-3	550	3	5% H_2/Ar
Fe-CNFs-550-4	550	4	5% H_2/Ar
Fe-CNFs-600-2	600	2	5% H_2/Ar
Fe-CNFs-650-2	650	2	5% H_2/Ar
